# Modifiable barriers to leisure-time physical activity during pregnancy: a qualitative study investigating first time mother’s views and experiences

**DOI:** 10.1186/s12884-015-0529-9

**Published:** 2015-04-22

**Authors:** Megan Connelly, Helen Brown, Paige van der Pligt, Megan Teychenne

**Affiliations:** Centre for Physical Activity and Nutrition Research, School of Exercise and Nutrition Sciences, Deakin University, 221 Burwood Hwy, Burwood, Vic 3125 Australia

**Keywords:** Pregnancy, Antenatal, Exercise, Correlates, Influences

## Abstract

**Background:**

Evidence suggests physical activity often declines during pregnancy, however explanations for the decline are not well understood. The aim of this study was to identify modifiable barriers to leisure-time physical activity among women who did not meet physical activity guidelines during pregnancy.

**Methods:**

Analyses were based on data from 133 mothers (~3-months postpartum) who were recruited from the Melbourne InFANT Extend study (2012/2013). Women completed a self-report survey at baseline in which they reported their leisure-time physical activity levels during pregnancy as well provided an open-ended written response regarding the key barriers that they perceived prevented them from meeting the physical activity guidelines during their pregnancy. Thematic analyses were conducted to identify key themes.

**Results:**

The qualitative data revealed six themes relating to the barriers of leisure-time physical activity during pregnancy. These included work-related factors (most commonly reported), tiredness, pregnancy-related symptoms, being active but not meeting the guidelines, lack of motivation, and a lack of knowledge of recommendations.

**Conclusion:**

Considering work-related barriers were suggested to be key factors to preventing women from meeting the physical activity guidelines during pregnancy, workplace interventions aimed at providing time management skills along with supporting physical activity programs for pregnant workers should be considered. Such interventions should also incorporate knowledge and education components, providing advice for undertaking leisure-time physical activity during pregnancy.

## Background

There is irrefutable evidence of the role that physical activity plays in the prevention of diseases such as cardiovascular disease, type 2 diabetes, hypertension, obesity and some cancers [1]. During pregnancy, however, physical activity has additional benefits such as a decreased risk of pre-eclampsia, excess gestational weight gain and gestational diabetes mellitus [2,3]. Further, leisure-time physical activity during pregnancy has been linked with mental health benefits such as reduced risk of antenatal depression [4]. Moreover, it has been found that those participating in physical activity early in pregnancy reported fewer symptoms of nausea and vomiting and have increased fitness and energy levels in later pregnancy [5].

Despite the well-documented health benefits to the mother and baby for women who undertake regular physical activity [6], it has been shown that compared to pre-pregnancy physical activity, women’s physical activity levels during pregnancy are often either reduced or ceased [7], in particular that of leisure-time physical activity [8]. Sports Medicine Australia have suggested that already active women without pregnancy complications should continue their exercise program after consultation with their doctor [9] and inactive pregnant women may begin an exercise program under doctors guidance [9]. Similarly, in the U.S it is recommended that in absence of obstetric complications, as per the adult guidelines, pregnant women should accumulate 30 minutes or more of moderate-intensity physical activity on most, and preferable all, days of the week [10,11]. Despite these guidelines, research has shown that only 32% of pregnant women in Australia meet the guidelines for physical activity [12], which is far lower than the general population (53%) [13]. These low rates of physical activity during pregnancy are concerning considering the evidence supporting the benefits of regular physical activity for both the mother and her child, as well as the fact that that these decreased levels of moderate-intensity physical activity have been show to persist well into the postpartum period [14].

In order to understand why a substantial proportion of women do not participate in regular physical activity during pregnancy, researchers need to examine barriers that women perceive to prevent them from engaging in antenatal leisure-time physical activity. Multiple studies have shown that non-modifiable correlates such as education, income, age and children [15-17] were associated with lower levels of leisure-time physical activity during pregnancy. While this information is appropriate for the identification of ‘at risk’ groups within this population, these are non-modifiable factors, which cannot be changed through application of intervention. Far fewer studies have investigated the modifiable correlates of leisure-time physical activity during pregnancy. Of the existing research, studies using focus groups amongst first-time [18] and non-first time pregnant women [19] have shown that factors such as low self-efficacy, pregnancy-related symptoms, lack of social support and knowledge were identified as barriers to leisure-time physical activity during pregnancy [18,19]. Open-ended questions were used to identify these barriers and included “Of all things that prevent you from exercising, which are the most powerful ones?” [19] and “What is the one main reason that keeps you from being more active while you are pregnant, either during work or nonworking time?” [18]. These barriers encompass the constructs of the Ecological Model, which posits that physical activity is influenced by intrapersonal (e.g. self-efficacy), interpersonal/social (e.g. social support) and physical environmental (e.g. access to facilities) factors [20]. Since the ecological model has been useful in guiding previous research into the understandings of correlates of physical activity in the general population [21], it may be appropriate to investigate the influences on leisure-time physical activity using such a model in other high-risk target groups such as pregnant women, although to date few studies have used this model in this target group.

Thus the aim of this study was to identify modifiable barriers to leisure-time physical activity among women who did not meet physical activity guidelines during pregnancy. It was hypothesized that barriers to leisure-time physical activity during pregnancy would encompass the constructs of the ecological model (i.e. include intrapersonal, interpersonal and physical environmental factors). Such information is important in order to help inform the development of physical activity interventions amongst pregnant women.

## Methods

### Study design and participants

The present study involved qualitative analysis from baseline survey data, collected as part of the InFANT (Infant Feeding, Activity and Nutrition Trial) Extend study that was conducted in 2012/2013. The InFANT Extend study was a randomized controlled trial, delivered to first-time parents, which aimed to test the effectiveness of providing parental support and knowledge regarding infant’s health behaviours (i.e. diet and physical activity) on early childhood (infant) health outcomes and behaviours. Data was collected from women who were on average 3-months postpartum, and who were recruited from existing first time mothers groups within Maternal and Child Health Centers, in seven local government areas throughout Victoria across a range (low, medium, high) of socioeconomic neighbourhoods.

In total, 531 women from 62 parent groups (28 = low, 20 = medium, 14 = high SEP) were approached to participate in the InFANT Extend study and 477 women (90%) agreed to participate. From the 475 women who completed the baseline survey, a total of 140 women reported not meeting the physical activity guidelines during pregnancy (this was defined as “during your pregnancy, did you undertake physical activity in your leisure-time which made you breathe harder for at least 150 minutes a week? For example 30 minutes a day, 5 days a week”). Of those, 133 (28%) women provided a qualitative response as to why they did not meet the guidelines and thus were included for analysis in this study.

### Procedures

This study was approved by the Deakin University Human Research Ethics Committee (EC Part 2-2007-175) and the Victorian Government Department of Human Services, Office for Children Research Co-ordinating Committee. After written consent was provided by women, participants completed questionnaires which were distributed by the researchers at the second recruitment visit to the Maternal and Child Health Centre. Women took the survey away with them and returned the completed survey in person at their first InFANT session, approximately 1 week later. If a mother was unable to make it to the first InFANT session she returned the completed survey via mail, using a reply paid envelope provided.

### Measures

The self-report survey included measures of demographic characteristics such as age, country of birth, marital status, education and occupation (post pregnancy). Further, whether women met the physical activity guidelines during pregnancy was measured in the survey using the following retrospective question: “During your pregnancy, did you undertake physical activity in your leisure-time which made you breathe harder for at least 150 minutes a week (e.g. 30 minutes a day, 5 days a week? (Includes brisk walking, swimming, jogging, dancing etc.)” Available responses were: ‘yes in every trimester’, ‘yes, but not in every trimester’, and ‘no’. Women who provided a ‘no’ response were asked to complete the next question, which asked women to provide the reasons as to why they did not undertake leisure-time physical activity during pregnancy that made them breathe harder over this time.

### Statistical analysis

Descriptive statistics (on demographic data) were analyzed using SPSS software (version 21). During this process participant identification was removed. For qualitative analyses, NVivo software was used to organize data and perform thematic analyses. Thematic analysis was chosen as it allows for flexibility and has been identified as an accessible tool for providing rich and detailed qualitative data [22,23]. The first author read the survey responses several times and notes were taken when relevant and reoccurring responses were made. Initially, participant responses were dichotomised according to low, medium or high socioeconomic position, which was determined by education level (categorised as did not complete high school (low); completed year high school or equivalent/trade/apprenticeship/certificate/diploma (medium); completed tertiary education (high)). Following this, data was coded using descriptive labels (i.e. advice, pregnancy complications, illness). Once responses were coded, themes were identified through linking and categorizing the individual codes together, (e.g. tiredness, work-related barriers, lack of motivation, knowledge, active but not meeting the guidelines) [22]. In order to assess the reliability of coding between researchers, coding was performed independently on a sub-sample of surveys (n = 10) by three separate authors [22]. The authors then met to discuss any discrepancies in coding, in which no discrepancies were evident.

The ecological model was used to guide analysis by categorizing the themes into either intrapersonal, interpersonal and physical environmental barriers [20]. Key themes (reflecting the main barriers to leisure-time physical activity during pregnancy) were determined using simple counts of how often these were mentioned by participants. In order to illustrate each theme, a sample of quotes was selected to provide a concise summary reflecting these. Pseudonyms were assigned to each participant in order to ensure anonymity.

A total of 27 codes were identified within the data. From this, nine themes were initially derived. However, two themes (‘already doing enough activity’ and ‘sickness’) were integrated into already existing themes (e.g. ‘sickness’ was integrated into either ‘pregnancy-related symptoms’ and ‘non-pregnancy related health barriers’). Finally, during the analysis one theme (‘non-pregnancy related health barriers’) was dropped due to only six women mentioning it.

## Results

Table 1 represents the demographic characteristics of the sample (n = 133), all of which were insufficiently active during leisure-time throughout each trimester of their pregnancy.

**Table 1:** **Demographic characteristics of first-time mothers not meeting the physical activity guidelines during pregnancy (n = 133)**

Characteristic	N	Percentage
Age	Mean age 31.9 years	
**Country of birth**		
Australia	110	83%
UK	4	3%
New Zealand	2	2%
Vietnam	2	2%
Other	15	11%
**Marital status**		
Married or defacto	128	96%
Separated widowed or divorced	1	<1%
Never married	4	3%
**Highest qualification**		
No formal or up to year 10 (low SEP)	7	5%
Year 12/trade apprentice/certificate diploma (med SEP)	61	46%
University degree or higher (high SEP)	65	49%
**Occupation status**		
Working full time	5	4%
Working part time	7	5%
Unemployed/laid off	1	<1%
Keeping house and/or raising children full time	119	90%
**Weekly household income**		
$1-$599 per week	40	35%
$600-$1499 per week	23	20%
$1500-$2000+ per week	8	7%
Other	45	39%

Six key themes emerged from the qualitative data, all of which lie within the intrapersonal construct of the ecological model [20]. These were: (i) work-related barriers (such as being too tired from work, not having enough time because of work duties or having a physically active job); (ii) tiredness (i.e. women often felt too tired to exercise); (iii) pregnancy-related barriers (i.e. these included morning sickness, muscle or joint pain and swelling and high risk pregnancies); (iv) being active but not meeting the guidelines (i.e. women often reported being physically active during pregnancy, however it was not to the recommended level); (v) lack of motivation (i.e. some women did not feel the need to exercise or just did not want to); (vi) lack of knowledge of recommendations (i.e. lack of personal knowledge or inadequate advice provided from a health professional to limit physical activity in women without complications).

### Work-related barriers (n = 48)

The most commonly reported barriers to leisure-time physical activity during pregnancy were work-related. Responses were similar across the different socioeconomic groups. There were three main reasons identified as to why work was a barrier. Firstly women felt they had a lack of time to be active due to work commitments, as reflected below;

*“Because after a full day at work & coming home to cook tea and do other jobs there weren’t enough hours in the day” (Mary, medium SEP)*

Secondly, women suggested that they felt too tired after work to be physically active, for example;

*“I worked 40 hrs a week in a supermarket until 34 weeks- I was very tired” (Susan, low SEP)*

Thirdly, several women reported having a physically active job, and assumed they were physically active enough at work.

*“I think I had enough physical activities at work to keep me healthy” (Jessica, high SEP)*

### Tiredness (n = 33)

Another barrier reported by a large proportion of women was that of feeling too tired to engage in physical activity. These responses were again mentioned by women in all socioeconomic groups. The key reason for being tired was reportedly due to work, although several women suggested their tiredness was in relation to the physical effects that pregnancy has on energy levels.

*“Was exhausted and not much energy” (Michelle, low SEP)*

### Pregnancy-related symptoms (n = 36)

A very common response for undertaking little to no leisure-time physical activity during pregnancy was due to morning sickness. These responses were mentioned by a third of the women, across all socioeconomic groups.

*“Severe/continued morning sickness throughout pregnancy” (Angela, high SEP)*

Other pregnancy-related symptoms included physical limitations such as pelvic and back pain due to or enhanced as a result of the pregnancy. Further, there were a small number of women who had been identified as having a high-risk pregnancy, and thus little to no physical activity was professionally advised.

*“I was pregnant with twins and these were IVF babies, so I was told no extra activity” (Tara, medium SEP)*

### Active, but not meeting the recommendations (n = 34)

It was often reported that women participated in leisure-time physical activities throughout their pregnancy; however, it was not to the levels recommended in the physical activity guidelines. For example;

*“I did just not that frequently. Maybe once or twice a week” (Kathy, high SEP)*

These responses were more frequently cited among women of a high socioeconomic position, when compared to those from low or middle socioeconomic positions.

### Lack of motivation (n = 19)

Several women suggested a lack of motivation when it came to physical activity during their pregnancy. These types of responses were more common from women of a low to medium socioeconomic position. Other women mentioned that they did little to no physical activity before pregnancy and subsequently did not do physical activity during pregnancy. Whilst a handful of women stated they did not like exercising or were simply too lazy to do it. For example;

*“I felt lazy whilst pregnant” (Joanna, medium SEP)*

### Lack of knowledge of recommendations (n = 10)

Several women reported having a lack of knowledge about physical activity during pregnancy such as not knowing if it was safe and not understanding that it was important. This was more frequently cited among those women from low to medium socioeconomic groups. For example;

*“Was not sure whether exercise was safe” (Rachel, medium SEP)*

*“Never got told to” (Sally, low SEP)*

Other women suggested that they had been advised by doctors to restrict physical activity, even though they did not report any pregnancy-related health complications.

*“Because [I] was unfit beforehand and was advised not to increase level of exercise if not used to it” (Carrie, high SEP)*

There were also further concerns about physical activity and the perception of causing harm to the baby as shown below,

*“Was scared I would over do it or harm my baby” (Samantha, high SEP)*

## Discussion

The aim of the current study was to identify modifiable barriers to leisure-time physical activity among women who did not meet physical activity guidelines during pregnancy. Research has provided sound evidence on the benefits of regular physical activity during pregnancy [2,3]. However, despite the well-documented benefits, there have been few studies that have focused on identifying the barriers to physical activity that women face during pregnancy.

The current study used the Ecological model [20] as a guide for the data analysis and to organize conclusions of the study. However, although the ecological model comprises of three constructs to explain behaviour change (intrapersonal, interpersonal and physical environmental factors), the present study found only intrapersonal barriers/themes as reasons for not engaging in regular leisure-time physical activity during pregnancy. Previous research has indicated that interpersonal (e.g. lack of role modelling and emotional support) and physical environmental (e.g. lack of access, weather) factors also contribute to explaining leisure-time physical inactivity during pregnancy [24]. However, consistent with our findings, other research has shown that the most commonly cited factors which influence leisure-time physical activity in pregnancy are intrapersonal barriers such as lack of time, tiredness or lower energy during pregnancy [8,25] a finding which was further highlighted by Evenson et al. who found that 85% of women reported an individual-level correlate (i.e. health reasons, lack of time/energy/knowledge/enjoyment) as their main barrier to exercise during pregnancy [18].

In the current study, the most commonly reported barriers to leisure-time physical activity during pregnancy were work-related factors. It was shown that women often reported having a lack of time due to work commitments, a lack of energy because of work, and perceiving that their job was already physically demanding which they felt contributed to meeting the physical activity guidelines. Although the total number of women who worked during pregnancy and the type of employment is not known for this sample, Australian national statistics estimate that over half of the pregnant women in Australia work during pregnancy [26]. Since a high portion of pregnant women in Australia work during pregnancy, antenatal physical activity interventions could look at targeting pregnant women in the workplace. Previous research has suggested that pregnant women need strategies to make it easier to be physically active at work [18], which is further supported by the findings of the current study. Furthermore, previous studies have further identified the workplace as an appropriate setting in which to deliver preventative health intervention programs to reach a wide selection of the adult population [27,28] and therefore similar strategies could be developed to help pregnant women in the workforce to engage in physical activity.

Another barrier to leisure-time physical activity during pregnancy that was frequently reported by women in the current study was tiredness/having a lack of energy. Reasons for the lack of energy were often cited as being due to work commitments or household duties, while others just mentioned feeling exhausted, which could be assumed due to the physiological changes women go through during pregnancy [29]. Consistent with previous research, feeling tired and having a lack of energy are the most commonly reported reasons for not being physically active during pregnancy [8,17,18,24,30]. Since it has been previously shown that regular physical activity is associated with increased fitness and energy levels of the mother during pregnancy [5], future programs may emphasize the energy-related benefits of physical activity for pregnant women, which they may not be aware of.

In contrast to previous research [18], pregnancy-related symptoms such as feeling ill/morning sickness were common barriers identified by a number of women in the current study. However, research has shown that women who are physically active in early pregnancy reported fewer symptoms such as nausea, vomiting and back pain in later pregnancy [5,15]. Thus, this information may be useful to provide to pregnant women as a motivational strategy to be more active if they do experience such symptoms. However, considering much of that research was cross sectional, future longitudinal and intervention research is needed to understand the direction of these relationships (i.e. whether physical activity reduces pregnancy-related symptoms, or whether those with pregnancy-related symptoms are less likely to engage in physical activity).

Our findings also indicate that knowledge (e.g. how to exercise safely whilst pregnant and physical activity recommendations during pregnancy) is an important factor that needs to be considered when developing interventions to promote physical activity during pregnancy. The current study revealed that a number of women were unclear on what the physical activity recommendations were during pregnancy or whether it was safe. This finding is similar to that of a previous qualitative study in which pregnant women mentioned receiving a lack of advice from health providers regarding physical activity, with some reporting to have not received any advice [18]. Since general practitioners and health professionals (e.g. antenatal health care professionals and midwives) have been identified as an ideal avenue in which to provide information regarding healthy lifestyle behavior’s for pregnant women [31], health professionals may be the key to enhancing pregnant women’s knowledge of the physical activity recommendations and benefits of activity during pregnancy [31].

Considering lack of knowledge was more frequently cited among those women from low to medium socioeconomic groups, future intervention strategies aimed at increasing pregnant women’s knowledge of physical activity recommendations and safety principles should specifically target these population groups.

Limitations of this study should be acknowledged. Firstly, the survey item assessing physical activity levels and the key barriers to physical activity during pregnancy was conducted retrospectively, with participants on average 3-months postpartum. Thus there is an increased chance of recall issues. Although the open-ended question allowed for emerging (rather than pre-defined) barriers to physical activity to be identified, there may have been additional barriers experienced by women, but not mentioned. Secondly, adherence to physical activity guidelines was based on leisure-time physical activity only, which may not have captured other activities performed by women such as work-related or domestic activity. Further, the terminology used to assess the physical activity guidelines (i.e. “breathe harder”) may be open to misinterpretation, however this terminology was based on that used in previously published and validated questionnaires [32]. Given that the study was cross-sectional, longitudinal research is warranted to assess whether women’s physical activity behaviour and influences change postpartum, an area which required further research [14,33]. Generalization of the study to the wider pregnancy population may be limited due to the over representation of women from a higher socioeconomic position (measured in terms of education). Further, there was a lack of information regarding women’s occupational status during pregnancy as well as pre-pregnancy physical activity, which may have been useful to contextualize findings. Barriers to physical activity among women who reported meeting the guidelines was not assessed and therefore differences in women’s barriers according to their physical activity levels (i.e. those meeting guidelines and those not) could not be presented, which may be an area of future interest. Moreover, the study recruited predominantly first-time mothers and since the barriers experienced by non-first-time mothers may be quite different, results may need to be confirmed in a broader sample of pregnant women for example including both first-time and non-first-time mothers. Finally a large proportion (63%) of women in the Infant Extend study reported meeting the physical activity guidelines during their pregnancy. This is in contrast to national statistics, which suggests that only 32% of pregnant women in Australia are meeting the physical activity guidelines during their pregnancy [12].

A key strength of this study was the application of the ecological model, which was used as a theoretical framework to guide the study design and data analysis [20]. The qualitative design of the study provided a rich and detailed insight into the barriers that women perceived to be preventing them from engaging in regular physical activity while pregnant, currently an understudied yet highly important research area. Further the large sample size (n = 133) ensured data saturation occurred for qualitative analysis.

## Conclusions

This research adds to the limited body of evidence regarding the modifiable barriers that women perceive to prevent them from being physically active during pregnancy. Intrapersonal barriers were highlighted as being particularly important for explaining leisure-time physical inactivity in this population group. The findings from this study may help to inform the development of intervention strategies aimed at promoting physical activity during pregnancy. Strategies could include provision of opportunities for physical activity during the workday such as walking meetings, group fitness activities during lunch breaks and providing information and guidance as to the type of moderate-intensity exercises, which can be performed during pregnancy. Further research needs to investigate the involvement that health practitioners can provide in terms of educating pregnant women (particularly those of low to medium socioeconomic position) regarding the physical activity recommendations and benefits during pregnancy including possible reduction in pregnancy-related symptoms.

## References

[1] Warburton DE, Nicol CW, Bredin SS (2006). Health benefits of physical activity: the evidence. Can Med Assoc J.

[2] Chasan-Taber L, Schmidt MD, Pekow P, Sternfeld B, Manson J, Markenson G (2007). Correlates of physical activity in pregnancy among Latina women. Matern Child Health J.

[3] Dempsey JC, Sorensen TK, Williams MA, Lee IM, Miller RS, Dashow EE (2003). A prospective study of gestational diabetes mellitus risk in relation to physical activity before and during pregnancy. Am J Obstet Gynecol.

[4] Shivakumar G, Brandon AR, Snell PG, Santiago-Munoz P, Johnson NL, Trivedi MH (2011). Antenatal depression: a rationale for studying exercise. Depress Anxiety.

[5] Morris SN, Johnson NR (2005). Exercise During Pregnancy: A critical appraisal of the literature. J Reprod Med.

[6] Doustan M, Seifourian M, Zarghami M, Azmsha T (2012). Relationship between Physical Activity of Mothers before and during Pregnancy with the Newborn Health and Pregnancy Outcome. J Phys Ed Sport.

[7] Borodulin K, Evenson K, Herring A (2009). Physical activity patterns during pregnancy through postpartum. BMC Womens Health.

[8] Symons Downs D, Hausenblas HA (2004). Women’s exercise beliefs and behaviors during their pregnancy and postpartum. J Midwifery Womens Health.

[9] Sport Medicine Australia (2001). Women in Sport: Fact Sheet No.2. Exercise in pregnancy.

[10] Artal R, O’Toole M (2003). Guidelines of the American College of Obstetricians and Gynecologists for exercise during pregnancy and the postpartum period. Br J Sports Med.

[11] American College of Obstetricians and Gynecologists (2003). Exercise during pregnancy and the postpartum period. Clin Obstet Gynecol.

[12] Wilkinson SA, Miller YD, Watson B (2009). Prevalence of health behaviours in pregnancy at service entry in a Queensland health service district. Aust N Z J Public Health.

[13] Australian Bureau of Statistics (2013). Australian Health Survey: Physical Activity, 2011–12.

[14] Pereira MA, Rifas-Shiman SL, Kleinman KP, Rich-Edwards JW, Peterson KE, Gillman MW (2007). Predictors of change in physical activity during and after pregnancy: Project Viva. Am J Prev Med.

[15] Foxcroft KF, Rowlands IJ, Byrne NM, McIntyre HD, Callaway LK (2011). Exercise in obese pregnant women: The role of social factors, lifestyle and pregnancy symptoms. BMC Pregnancy Childbirth.

[16] Gaston A, Vamos C (2013). Leisure-time physical activity patterns and correlates among pregnant women in Ontario, Canada. Matern Child Health J.

[17] Jukic AMZ, Evenson KR, Herring AH, Wilcox AJ, Hartmann KE, Daniels JL (2012). Correlates of Physical Activity at Two Time Points During Pregnancy. J Phys Act Health.

[18] Evenson KR, Moos M-K, Carrier K, Siega-Riz AM (2009). Perceived barriers to physical activity among pregnant women. Matern Child Health J.

[19] Marquez DX, Bustamante EE, Bock BC, Markenson G, Tovar A, Chasan-Taber L (2009). Perspectives of Latina and Non-Latina White Women on Barriers and Facilitators to Exercise in Pregnancy. Women Health.

[20] McLeroy KR, Bibeau D, Steckler A, Glanz K (1988). An ecological perspective on health promotion programs. Health Educ Q.

[21] Trost S, Owen N, Bauman AE, Sallis JF, Brown W (2002). Correlates of adults’ participation in physical activity: review and update. Med Sci Sports Exerc.

[22] Green J, Willis K, Hughes E, Small R, Welch N, Gibbs L (2007). Generating best evidence from qualitative research: the role of data analysis. Aust N Z J Public Health.

[23] Braun V, Clarke V (2006). Using thematic analysis in psychology. Qual Res Psychol.

[24] Leiferman J, Swibas T, Koiness K, Marshall JA, Dunn AL (2011). My Baby, My Move: Examination of Perceived Barriers and Motivating Factors Related to Antenatal Physical Activity. J Midwifery Womens Health.

[25] Symons Downs D, Chasan-Taber L, Evenson KR, Leiferman J, Yeo S (2012). Physical Activity and Pregnancy: Past and Present Evidence and Future Recommendations. Res Q Exerc Sport.

[26] Australian Bureau of Statistics (2011). Physical Activity in Australia: A Snapshot, 2007–08.

[27] Anderson LM, Quinn TA, Glanz K, Ramirez G, Kahwati LC, Johnson DB (2009). The effectiveness of worksite nutrition and physical activity interventions for controlling employee overweight and obesity a systematic review. Am J Prev Med.

[28] Tan AM, LaMontagne AD, Sarmugam R, Howard P (2013). A cluster-randomised, controlled trial to assess the impact of a workplace osteoporosis prevention intervention on the dietary and physical activity behaviours of working women: study protocol. BMC Public Health.

[29] McMurray RG, Mottola MF, Wolfe LA, Artal R, Millar L, Pivarnik JM (1993). Recent advances in understanding maternal and fetal responses to exercise. /Progres recents dans la comprehension des reponses maternelles et foetales a l ’ exercice. Med Sci Sports Exerc.

[30] Duncombe D, Wertheim EH, Skouteris H, Paxton SJ, Kelly L (2009). Factors related to exercise over the course of pregnancy including women’s beliefs about the safety of exercise during pregnancy. Midwifery.

[31] van der Pligt P, Campbell K, Willcox J, Opie J, Denney-Wilson E (2011). Opportunities for primary and secondary prevention of excess gestational weight gain: General Practitioners’ perspectives.. BMC Fam Pract.

[32] Craig CL, Marshall AL, Sjostrom M, Bauman AE, Booth ML, Ainsworth BE (2003). International physical activity questionnaire: 12-country reliability and validity. Med Sci Sports Exerc.

[33] Evenson KR (2011). Towards an Understanding of Change in Physical Activity from Pregnancy Through Postpartum. Psychol Sport Exerc.

